# Supplementary material for: A high-throughput method for determining the amylose content of rice
Source: PLoS One. 2025 Nov 25;20(11):e0336600. doi: 10.1371/journal.pone.0336600 (PMC12646410; doi:10.1371/journal.pone.0336600)
Supplement: Attachment — Submitted filename: Reviewers comment on PONE-D-24-60263.docx [file pone.0336600.s005.docx]

**Reviewer’s comment on PONE-D-24-60263**

**General comment:**

The article presents an innovative high throughput method of measuring amylose analysis in rice samples. However, the manuscript was not written in clear, concise and readable English. The advantage/importance of the new method was not clearly presented in the manuscript due to unclear presentation of the materials and method section.

However, I will advise a complete English editing of the manuscript and that the material and method section should be re-written. It was presented as if the authors were writing step by step procedure in a laboratory manual.

**Abstract**

The abstract should be re-written to follow this pattern:

1. Introductory statement
2. Problem statement
3. Objective of study/statement of objective
4. Materials and method
5. Results and discussion
6. Conclusion

**Introduction**

There was no introduction section, I only see’ information’ is this the journal’s style?

Line 33-34: clumsy statement, not clear, please revise

Line 47: use ‘defatting instead of ‘degreasing’

Line 57: what is AAC? Write in full

Line 61: ‘it is’ instead of ‘it’s’

Line 63: “due to the fact that’

Materials and Methods

**Materials & Method**

**There must be complete revision of this section to bring out the beauty of the manuscript.**

**Results, Discussion, Conclusion**

Table 3 is not clear

Because of the materials and method section that is muddled up, the beauty, or advantage of the new method is not well elucidated.

The authors highlighted the advantages of the new method, but the methods used to develop the new method is not clearly presented.
