# Supplementary material for: A high-throughput method for determining the amylose content of rice
Source: PLoS One. 2025 Nov 25;20(11):e0336600. doi: 10.1371/journal.pone.0336600 (PMC12646410; doi:10.1371/journal.pone.0336600)
Supplement: Attachment — Submitted filename: Reviews.docx [file pone.0336600.s006.docx]

This article introduces an improved high-throughput method for determining branched starch content based on the national standard. The research findings indicate that the chemical values measured using this method do not exhibit significant differences compared to those obtained through the national standard method. However, several issues remain in the article, which require clarification from the authors.

Suggested Improvements:

(1) Single-Grain Analysis vs. Bulk Sample Analysis

Literature has suggested that conducting rice quality analysis at the single-grain level is more conducive to reducing the breeding cycle of rice. However, the method described in this article processes bulk rice samples to obtain polished rice flour. It is recommended that the authors add an experimental group that processes individual or a few rice grains using the proposed method. The results should then be compared with those obtained using the SDM method to enhance the reliability of the data presented.

**References:**

L.E. Agelet, C.R. Hurburgh, Limitations and current applications of Near Infrared Spectroscopy for single seed analysis, Talanta, 121 (2014) 288‒299.

(2) Optimization of the SSDM Method

The article claims that the SSDM method is superior to the SDM method. Unfortunately, no schematic or flowchart is provided to illustrate how the method has been optimized. It is recommended that the authors include a logical explanation of the optimization process. Additionally, the description of experimental procedures is overly detailed. Unnecessary steps, such as the sample preparation process in the SDM method, should be removed to better highlight the innovative aspects of the study.

(3) Limited Sample Size

In the "Materials and Methods" section, it is mentioned that only six rice samples were analyzed using both the SDM and SSDM methods, which lacks sufficient representativeness. It is recommended to expand the sample size, including both indica and japonica rice varieties, to improve the generalizability of the SSDM method.

(4) Comparison with Existing Single-Grain Measurement Techniques

Numerous studies have reported well-established single-grain rice measurement methods, including chemical and spectroscopic techniques. The authors should compare their method with these existing techniques to demonstrate its novelty and advantages.

**References:**

Wu J G, Shi C H. Prediction of grain weight, brown rice weight and amylose content in single rice grains using near-infrared reflectance spectroscopy[J]. Field Crops Research, 2004, 87(1): 13-21.

Yang, J.H., 1992. Study on test methodology of amylose content of single (half) seed of rice. Crop Variety Res. 1, 33–35.

Dai J, Zhang H, Zhu Y, et al. A high–throughput method for determination of apparent amylase content in rice. Acta Agriculturae Zhejiangensis，2014, 26(6): 1421–1424.

Fan S, Xu Z, Cheng W, Wang Q, Yang Y, Guo J, Zhang P, Wu Y. Establishment of Non-Destructive Methods for the Detection of Amylose and Fat Content in Single Rice Kernels Using Near-Infrared Spectroscopy. *Agriculture*. 2022; 12(8):1258.

(5) Clarification on the Starch-Iodine Absorption Spectrum (Lines 128–145)

The phrase "starch-iodine" should be corrected to "amylose-iodine."

The article does not specify which wavelength was used for absorbance measurements in the subsequent experiments, which may cause confusion. The authors should clarify this information.

Placement of Results within the Discussion Section (Lines 149–157)

This section presents research results, which should not be included in the discussion. It is suggested that this part be removed or relocated appropriately.

(6) Significance Testing in Correlation Analysis (Line 169)

The correlation analysis between the two methods should include significance test results in the figures or tables to confirm that the differences between the results obtained by both methods are not statistically significant. This would provide a stronger basis for subsequent discussions.

(7) Precision Analysis of the SSDM Method (Line 193, Table 1)

The authors should discuss differences in measurement precision among various rice varieties (e.g., indica and japonica) under different digestion times and sample amounts.

(8) Effect of Digestion Time on SSDM Accuracy (Line 197, Table 2)

Standard samples should be used to determine the effect of digestion time on SSDM measurement accuracy, as the amylose content of standard samples is already known.

(9) Language and Formatting Issues:

The article contains multiple grammatical errors and redundancies, and it appears that no thorough proofreading has been performed. Examples include:

Line 193: "table1" should be written as "Table 1."

Line 12:

Original: "The simple high-throughput determination method for amylose content is an improved method based on the national standard (GB/T15683-2008/ISO6647-1:2007)."

Suggested Revision: "The improved high-throughput amylose determination method is based on the national standard (GB/T15683-2008/ISO6647-1:2007)."

Line 20:

Original: "There is no significant difference between the amylose content measured using this method and the values obtained through the national standard method."

Suggested Revision: "The amylose content measured by this method shows no significant difference from that obtained through the national standard method."

There are numerous other instances of similar issues that require careful revision.

(10) Some of the references are outdated and should be replaced with more recent literature.

Conclusion:

The article presents valuable findings, but several aspects require improvement. The authors are encouraged to revise the manuscript based on the provided suggestions and refine the language for clarity and coherence.
