# Supplementary material for: A high-throughput method for determining the amylose content of rice
Source: PLoS One. 2025 Nov 25;20(11):e0336600. doi: 10.1371/journal.pone.0336600 (PMC12646410; doi:10.1371/journal.pone.0336600)
Supplement: Attachment — Submitted filename: Responses to Editors and reviewers.docx [file pone.0336600.s008.docx]

**Responses to Academic Editor's comments**

Thanks for your wonderful comments and constructive proposals. The reviewer points out that “The advantage/importance of the new method was not clearly presented in the manuscript due to unclear presentation of the materials and method section.” We have read through the manuscript and made some changes in this, for example, we have revised these sections of the manuscript accordingly, which can be found, and we have deleted a paragraph on page 5. And we also re-organized the paragraphs in page2, lines 33 to 34 as follows: “The texture and sensory quality of the rice are affected by the ratio of amylose to amylopectin. Usually, rice with high amylose content (AC) tends to have a harder texture, with the grains that are fluffy and easily dispersed. On the contrary, rice with a good taste, is soft and elastic, with shiny grains. That are not easy to be separated.”

The purpose of the research introduced in this article is ‌to evaluate‌ three *indica* rice varieties with varying amylose contents and three *japonica* rice varieties ‌by comparing‌ the SDM (the national standard method) and SSDM (the improved high-throughput measurement method). ‌The study aims to explore‌ the feasibility of optimizing the SSDM ‌built upon‌ the SDM framework, ‌with a focus on improving‌ measurement accuracy. ‌To enhance experimental efficiency‌, an innovative enzyme-linked immunosorbent assay (ELISA) was incorporated into the SSDM protocol. ‌This modification‌ not only ‌streamlined‌ the determination process but also ‌reduced operational costs‌. Additionally, ‌the study analyzed‌ the accuracy and ‌methodological limitations‌ of SSDM during implementation, ‌identifying factors‌ affecting its performance. ‌These innovations and findings‌ address gaps in prior studies on amylose quantification, ‌demonstrating‌ the advantages and application potential of SSDM in cereal science research.

**Reviewer’s comment on PONE-D-24-60263**

**RESPONSES TO REVIEWER #1**

**General comment:**

This article introduces an improved high-throughput method for determining branched starch content based on the national standard. The research findings indicate that the chemical values measured using this method do not exhibit significant differences compared to those obtained through the national standard method. However, several issues remain in the article, which require clarification from the authors.

**Suggested Improvements:**

1. **Single-Grain Analysis vs. Bulk Sample Analysis**

Literature has suggested that conducting rice quality analysis at the single-grain level is more conducive to reducing the breeding cycle of rice. However, the method described in this article processes bulk rice samples to obtain polished rice flour. It is recommended that the authors add an experimental group that processes individual or a few rice grains using the proposed method. The results should then be compared with those obtained using the SDM method to enhance the reliability of the data presented.

**References:**

L.E. Agelet, C.R. Hurburgh, Limitations and current applications of Near Infrared Spectroscopy for single seed analysis, Talanta, 121 (2014) 288‒299.

**Response:** This is a ‌valuable‌ suggestion‌. We ‌had‌ considered such materials in the experimental design‌. However, ‌since‌ the starch content trait in rice endosperm ‌is classified as‌ an endosperm-specific characteristic following triploid genetic laws‌, previous studies ‌have‌ discussed ‌this aspect‌‌. ‌Given‌ the gene dosage effect ‌observed in single grains or small samples of rice‌, the sample reproducibility ‌was‌ poor‌. Therefore, ‌rice noodles (mixed samples)‌ of known varieties ‌were‌ selected for the experiment ‌to avoid‌ single-grain rice as material, ‌thereby minimizing‌ errors caused by ‌gene dosage effects‌ in the endosperm. ‌We plan to‌ further explore analytical methods for single-grain rice in future work‌.

**References:**

Zhang AH, Xu CW, Mo HD, Genetic expression of several quality traits in *indica*-*japonica* hybrids. Acta Agronomica Sinica 1999, 25, 401-407.

Zhang T, Wang ZR, Mao XC, *et al*. Research progress of genes affecting rice grain quality. J. Anhui Agric. Sci. 2023, 51, 17-22.

2. **Optimization of the SSDM Method**

The article claims that the SSDM method is superior to the SDM method. Unfortunately, no schematic or flowchart is provided to illustrate how the method has been optimized. It is recommended that the authors include a logical explanation of the optimization process. Additionally, the description of experimental procedures is overly detailed. Unnecessary steps, such as the sample preparation process in the SDM method, should be removed to better highlight the innovative aspects of the study.

**Response:** Thank you to the reviewers for their valuable suggestions. We have revised the section of the manuscript section accordingly, which can be found on page 3, lines 89-108.

3. **Limited Sample Size**

In the "Materials and Methods" section, it is mentioned that only six rice samples were analyzed using both the SDM and SSDM methods, which lacks sufficient representativeness. It is recommended to expand the sample size, including both indica and japonica rice varieties, to improve the generalizability of the SSDM method.

**Response:** This is a valuable suggestion.‌ We initially considered such materials during experimental design. ‌However, because the *Waxy* gene regulates starch content in rice endosperm‌ a trait classified as triploid-inherited endosperm characteristics‌, its mechanism has been extensively documented in prior studies. For instance, Shao et al. reported that many Waxy alleles have been identified in rice, which are *Wx^a^*, *Wx^b^*, *Wx^in^*, *Wx^op^*, *Wx^mq^*, *Wx^mw^*, *Wx^lv^*, and *wx*. The AC of alleles decreases in the following, *Wx^a^* (20–25%), *Wx^b^* (15–18%) and *wx* (AC<2%). ‌To avoid interference with established genetic patterns‌, this experiment selected six representative common rice varieties for comparing SDM and SSDM characteristics. Looking ahead, we will place more emphasis on enhancing the experimental prototype. We will apply this experimental design in future research work.

4. **Comparison with Existing Single-Grain Measurement Techniques**

Numerous studies have reported well-established single-grain rice measurement methods, including chemical and spectroscopic techniques. The authors should compare their method with these existing techniques to demonstrate its novelty and advantages.

**References:**

Wu J G, Shi C H. Prediction of grain weight, brown rice weight and amylose content in single rice grains using near-infrared reflectance spectroscopy [J]. Field Crops Research, 2004, 87(1): 13-21.

Yang, J.H., 1992. Study on test methodology of amylose content of single (half) seed of rice. Crop Variety Res. 1, 33–35.

Dai J, Zhang H, Zhu Y, et al. A high–throughput method for determination of apparent amylase content in rice. Acta Agriculturae Zhejiangensis，2014, 26(6): 1421–1424.

Fan S, Xu Z, Cheng W, Wang Q, Yang Y, Guo J, Zhang P, Wu Y. Establishment of Non-Destructive Methods for the Detection of Amylose and Fat Content in Single Rice Kernels Using Near-Infrared Spectroscopy. *Agriculture*. 2022; 12(8):1258.

**Response:** This is a very good experimental design idea. We concur with the reviewer that additional studies, data, or further experimental results would be beneficial. Unfortunately, the laboratory does not have the necessary optical measuring instruments. On the other hand, due to SSDM being optimized based on the SDM method.

5. **Clarification on the Starch-Iodine Absorption Spectrum (Lines 128–145)**

The phrase "starch-iodine" should be corrected to "amylose-iodine."

The article does not specify which wavelength was used for absorbance measurements in the subsequent experiments, which may cause confusion. The authors should clarify this information.

**Response:** We apologize for causing confusion to the reviewer. In the original manuscript, the discussion regarding ‘Amylose-iodine absorption spectrum’ indicates the selection of experimental testing wavelengths. To offer a more explicit understanding, in the revised manuscript, the corresponding text has been reorganized and modified. It can be located on page 4, lines 142-162.

6. **Placement of Results within the Discussion Section (Lines 149–157)**

This section presents research results, which should not be included in the discussion. It is suggested that this part be removed or relocated appropriately.

**Response:** This section has been revised in the article, which this part removed. The removed content can be located on page 5.

7. **Significance Testing in Correlation Analysis (Line 169)**

The correlation analysis between the two methods should include significance test results in the figures or tables to confirm that the differences between the results obtained by both methods are not statistically significant. This would provide a stronger basis for subsequent discussions.

**Response:** This section has been revised in the article, which can be located on page 5, lines 174-177. The correlation analysis between the two methods should include significance test results in the figures or tables to confirm that the differences between the results obtained by both methods are not statistically significant. This would provide a stronger basis for subsequent discussions.

8. **Precision Analysis of the SSDM Method (Line 193, Table 1)**

The authors should discuss differences in measurement precision among various rice varieties (e.g., indica and japonica) under different digestion times and sample amounts.

**Response:** This section has been revised in the article, which can be located on page 5, lines 200-203. The trend in different rice varieties remains consistent across digestion times and sample weights. However, the *indica* rice varieties exhibit a larger coefficient of variation compared to the *japonica* rice varieties, resulting in less stable measurements. This instability may be linked to genetic differences between the two subspecies.

9. **Effect of Digestion Time on SSDM Accuracy (Line 197, Table 2)**

Standard samples should be used to determine the effect of digestion time on SSDM measurement accuracy, as the amylose content of standard samples is already known.

**Response:** This is a very good experimental design idea. This section ‌establishes‌ the feasibility of the SSDM method (Figs. 2 and 3), ‌followed by‌ analyzing the influencing factors‌. ‌The reliability of this method in practical applications is thereby confirmed. ‌Furthermore‌, the relationship between digestion time and standard sample reliability ‌has been extensively documented in prior research‌, ‌thus obviating the need for further elaboration.

10. **Language and Formatting Issues:**

The article contains multiple grammatical errors and redundancies, and it appears that no thorough proofreading has been performed. Examples include:

1) **Line 193: "table1" should be written as "Table 1."**

**Response:** I am sorry for my careless mistakes. As suggested by the reviewer, has been corrected in the revised manuscript, as seen in page 7, lines 208.

2) **Line 12:**

Original: "The simple high-throughput determination method for amylose content is an improved method based on the national standard (GB/T15683-2008/ISO6647-1:2007)."

Suggested Revision: "The improved high-throughput amylose determination method is based on the national standard (GB/T15683-2008/ISO6647-1:2007)."

**Response:** This is a very good idea. This section has been revised in the article. As suggested by the reviewer, has been corrected in the revised manuscript, as seen in page 2, lines 4-5.

3) **Line 20:**

Original: "There is no significant difference between the amylose content measured using this method and the values obtained through the national standard method."

Suggested Revision: "The amylose content measured by this method shows no significant difference from that obtained through the national standard method."

There are numerous other instances of similar issues that require careful revision.

**Response:** Thank you very much for the suggestions from the reviewing experts. This section has been revised in the article, as seen in page 2, lines 42-43.

11. **Some of the references are outdated and should be replaced with more recent literature.**

**Response:** We totally agree with the reviewer. In the revised manuscript, they have been added as the references as seen in pages 10-12, lines 293-355, meanwhile the other related references have also been referred.

Conclusion:

The article presents valuable findings, but several aspects require improvement. The authors are encouraged to revise the manuscript based on the provided suggestions and refine the language for clarity and coherence.

**RESPONSES TO REVIEWER #2**

**The comments are as follows:**

The article presents an innovative high throughput method of measuring amylose analysis in rice samples. However, the manuscript was not written in clear, concise and readable English. The advantage/importance of the new method was not clearly presented in the manuscript due to unclear presentation of the materials and method section.

However, I will advise a complete English editing of the manuscript and that the material and method section should be re-written. It was presented as if the authors were writing step by step procedure in a laboratory manual.

1. **Abstract**

The abstract should be re-written to follow this pattern:

1. Introductory statement
2. Problem statement
3. Objective of study/statement of objective
4. Materials and method
5. Results and discussion
6. Conclusion

**Response:** Thank you to the reviewers for their valuable suggestions. We have revised the Abstract section of the manuscript accordingly, which can be found in page 1, lines 29-45.

2. **Introduction**

There was no introduction section, I only see’ information’ is this the journal’s style?

**Response:** We sincerely apologize for the oversight, as we failed to verify the relevant information in the author's instructions.  As suggested by the reviewer, has been corrected in the revised manuscript. This section has been revised in the article, as seen in page 2, line 46.

3. **Line 33-34: clumsy statement, not clear, please revise**

**Response:** Thank you for the suggestions from the reviewing experts. We will include the clubsy statement for Line 33-34. The 'not clear' section has been modified. In the revised manuscript, the corresponding explanation and conclusion have been added, which can be found in page 2, lines 52-55 and given below: The texture and sensory quality of the rice are affected by the ratio of amylose to amylopectin. Usually, rice with high amylose content (AC) tends to have a harder texture, with the grains that are fluffy and easily dispersed. On the contrary, rice with a good taste, is soft and elastic, with shiny grains. That are not easy to be separated.

4. **Line 47: use ‘defatting instead of ‘degreasing’**

**R**esponse: Thank you to the reviewers for their valuable suggestions. We have made revisions to the inaccurate wording section of the manuscript accordingly, which can be found in page 2, line 54 and given below: ‘In addition, different types of national standards are derived based on whether the test sample is degreased’ had been instead of ‘In addition, different types of national standards are derived based on whether the test sample has been defatted’.

5. **Line 57: what is AAC? Write in full**

**Response:** This section has been revised in the article, which can be found in page 3, line 78 and given below: the apparent amylose content was abbreviated as AAC.

6. **Line 61: ‘it is’ instead of ‘it’s’.**

**Response:** Thank you to the reviewers for their valuable suggestions. This section has been revised in the article, which can be found in page 3, line 81.

7. **Line 63: “due to the fact that”**

**Response:** This section has been revised in the article, which can be found in page 3, lines 83-85.

8. **Materials and Methods**

There must be complete revision of this section to bring out the beauty of the manuscript. Results, Discussion, Conclusion

**Response:** Thank you to the reviewers for their valuable suggestions. This section has been revised in the article. We have made revisions to the section of the manuscript accordingly.

9. **Table 3 is not clear**

**Response:** Table 3 presents the accuracy analysis results of the *amylose content* determined by two methods. The results demonstrate that the SSDM method is relatively accurate and exhibits a low coefficient of variation. These findings provide a theoretical basis for implementing the SSDM method. *‌*This can be found in page 9, lines 218-225.

10. **Because of the materials and method section that is muddled up, the beauty, or advantage of the new method is not well elucidated. The authors highlighted the advantages of the new method, but the methods used to develop the new method is not clearly presented.**

**Response:** Thank you to the reviewers for their valuable suggestions. This section has been revised in the article. We have made revisions to the materials and method section of the manuscript accordingly, which can be found in page 3, lines 88-140.
