# Supplementary material for: A high-throughput method for determining the amylose content of rice
Source: PLoS One. 2025 Nov 25;20(11):e0336600. doi: 10.1371/journal.pone.0336600 (PMC12646410; doi:10.1371/journal.pone.0336600)
Supplement: Attachment — Submitted filename: Response to Reviewers 20250723.docx [file pone.0336600.s009.docx]

**Responses to Academic Editor's comments**

I sincerely thank you and the two reviewers for their comments and put forward very constructive suggestions and opinions to us. We have carefully reviewed and revised the manuscript. We believe that under the guidance of our beloved editors and review experts, we can give good and appropriate responses to all the comments listed below through our careful modification. The following is a list of major changes based on the opinions of editors and reviewers:

1. According to the comments of editors and reviewers, we highlighted the purpose of the research in the manuscript to facilitate readers' understanding of our work.
2. According to the reviewer's comments, we modified and corrected the content and wording of the manuscript.
3. We have carefully revised the manuscript, including correcting typing errors, adding content to explain the data in the chart, and modifying all texts according to the concerns of the reviewers. Please refer to the main revised manuscript for details.

Through our careful efforts to revise and improve, we hope that the revised version can meet the requirements and make the manuscript published in the form of *PloS One*. Thank you again for your wonderful comments and your valuable time for our manuscript. We sincerely look forward to seeing our working manuscript in your journal.

**Reviewer’s comment on PONE-D-24-60263R1**

**Responses to Academic Editors**

**Comments and Suggestions for Authors**

**1. Please address the following comments in order to make the final decision for your manuscript.**

**Response:** Thank you to the editors and reviewers for their comments and suggestions. We actively and seriously revise.

**2. The study addresses a relevant challenge in rice quality evaluation by proposing an improved high-throughput method for determining amylose content (SSDM), building upon an existing standard.**

**Response:** Thank you for the affirmation from the review experts. We have provided analysis and elaboration on page 2, lines 32-41, and also added Figure 1 and Table 4 to better illustrate the advantages and shortcomings of the improved SSDM method.

**3. The novelty of the SSDM method is moderately articulated. While the authors claim operational efficiency and reduced labor, the mechanistic or methodological innovation beyond equipment change and protocol condensation is insufficiently developed. The authors should clarify what is scientifically novel beyond throughput or procedural efficiency.**

**Response:** Thank you to the review experts for their suggestions and opinions. I believe these suggestions and opinions will contribute to a more scientific presentation of our research work. We have made further revisions on page 11, line 295 of the manuscript. The innovation of the experiment is that, the use of standard samples and the proposal of absorption peak schemes for the determination of amylose content have solved the problem of experimental repeatability caused by differences in reagents or experimenters in the article. In previous work, there was no prior reporting.

**4. The experimental design is limited by a very small sample size (six rice varieties), reducing the generalizability and statistical power of the conclusions. The authors must justify the small sample size or expand it. Include rationale for the selection of the specific varieties.**

**Response:** Thank you to the review experts for their suggestions and opinions. I believe these suggestions and opinions will contribute to a more scientific presentation of our research work. We have made further revisions on page 11, line 268 of the manuscript.

Genetic differences are internal factors affecting the content of amylose, and genes related to amylose content mainly include *Wx*, *dull*, and *floury*. Among them, *Wx* is the main gene that controls amylose content, located on the short arm of chromosome 6. The non-glutinous gene *wx* exhibits incomplete dominant inheritance of the glutinous gene *Wx*, and there is a significant dose-response relationship. *Wx* alleles have been reported, including *Wx^a^*, *Wx^b^*, *Wx^lv^*, *Wx^in^*, *Wx^op^*, *Wx^mp^*, and *wx*. These alleles can be distinguished by six functional polymorphic loci, namely Int1-1, Ex2-112, Ex4-53, Ex4-77, Ex6-62, and Ex10-115 (Fig. 1 in this manuscript). Due to the different phenotypes of allelic variations, the amylose content of also varies accordingly. In order to determine the relationship between these alleles, this is a valuable suggestion. We initially considered such materials during experimental design. However, because the Waxy gene regulates starch content in rice endosperm a trait classified as triploid-inherited endosperm characteristics, its mechanism has been extensively documented in prior studies. For instance, Shao et al. reported that many Waxy alleles have been identified in rice, which are *Wx^a^*, *Wx^b^*, *Wx^in^*, *Wx^op^*, *Wx^mq^*, *Wx^mw^*, *Wx^lv^*, and *wx*. The AC of alleles decreases in the following, *Wx^a^* (20–25%), *Wx^b^* (15–18%) and wx (AC<2%). Zhang et al. used the *Wx* gene sequence alignment method to sequence and analyze a large number of rice germplasm resources. The results indicate that *Wx^lv^* exists in most wild rice varieties. All *Wx* alleles have a cytosine (abbreviated as C) at the Ex10-115 and are orthologous with genes encoding GBSSI in other monocotyledonous plants.

An evolutionary tree for the *Wx* allele was constructed using sequences from some cultivated rice varieties. The ancestral *Wx* allele gave rise to the *Wx^lv^* gene, which diversified into distinct haplotypes，such as *Wx^lv^* I, II, III, and IV, through artificial selection. Research confirms that functional locus variations at Int1-1 and Ex6-62 classify *Wx^b^* and *Wx^in^* in *japonica* rice subspecies as *Wx^lv^* I. In contrast, *Wx^a^* and *Wx^op^* underwent base substitutions at Ex10-115 and Ex4-77, categorizing them as *Wx^lv^* II and IV types. *Wx^mp^* and *wx* are derivatives of *Wx^b^*. A 23 bp sequence deletion in the *wx* results in loss of *Wx* function, producing 0% amylose content and a waxy phenotype. These genetic differences highlight the locus's pivotal role in *Wx* evolution, supporting molecular design breeding for rice with specific amylose profiles and eating quality characteristics. In addition, at least 7 different *dull* have been discovered, with *du-1* and *du-4*, *du*-2120 and *du*-2035, located on different chromosomes, respectively. To avoid interference with established genetic patterns, this experiment selected 6 representative rice varieties for comparing SDM and SSDM characteristics. Looking ahead, we will place more emphasis on enhancing the experimental prototype. We will apply this experimental design in future research work.

**Figure 1.** Genotypes and phenotypes of different *Wx* alleles. (A) The structure of *Wx* gene (*Os06g0133000, LOC_Os06g04200*) and (B) corresponding apparent amylose content (AAC) in rice.

**References**

Shao, Y.; Peng, Y.; Mao, B.; Lv, Q.; Yuan, D.; Liu X. Allelic variations of the *Wx* locus in cultivated rice and their use in the development of hybrid rice in China. *PloS One.* 2020, *15*, e0232279

Zhang C, Zhu J, Chen S, Fan X, Li Q, Lu Y, Wang M, Yu H, Yi C, Tang S, Gu M, Liu Q. *Wx^lv^*, the ancestral allele of rice *Waxy* gene. *Mol Plant*. 2019, *12*, 1157-1166.用

Zhang AH, Xu CW, Mo HD, Genetic expression of several quality traits in *indica*-*japonica* hybrids. *Acta Agronomica Sinica* 1999, 25, 401-407.

Zhang T, Wang ZR, Mao XC, *et al*. Research progress of genes affecting rice grain quality. J. Anhui Agric. Sci. 2023, 51, 17-22.

**5. The comparison to the standard method lacks critical methodological controls, particularly regarding standardization of sample preparation and instrument calibration across both methods. Provide a detailed explanation of how comparability was ensured.**

**Response:** Unfortunately, I am unable to answer this question. The goal of experimental design is to optimize some limiting factors in the original method, such as the relatively simple and cumbersome selection of instruments and vessels in the SDM scheme, and the relatively large amount of experimental samples used. Although, this method is still used in rice quality identification research (added to the literature), its high sample demand poses a significant constrain for early generation screening with limited germplasm in genetic breeding. Notably, the SDM method is an industry standard with certain technical guidance in the determination of amylose content. Consequently, alternative standardized approaches were not considered for our study.

**References:**

Zhang T, Wang ZR, Mao XC, *et al*. Research progress of genes affecting rice grain quality. *J. Anhui Agric. Sci.* 2023, *51*, 17-22.

Shao, Y.; Peng, Y.; Mao, B.; Lv, Q.; Yuan, D.; Liu X. Allelic variations of the *Wx* locus in cultivated rice and their use in the development of hybrid rice in China. *PloS One.* 2020, *15*, e0232279.

Zhang C, Zhu J, Chen S, Fan X, Li Q, Lu Y, Wang M, Yu H, Yi C, Tang S, Gu M, Liu Q. *Wx^lv^*, the ancestral allele of rice *Waxy* gene. *Mol Plant*. 2019, *12*, 1157-1166.用

Chen, C.; Zhang, Y.F.; Wang, S.H. Study on the determination of amylose content of single rice grain. *Journal of Anhui Agri. Sci.* 2005, *33*, 196-197.

Zhang AH, Xu CW, Mo HD, Genetic expression of several quality traits in *indica*-*japonica* hybrids. *Acta Agronomica Sinica* 1999, 25, 401-407.

**6. Statistical rigor is compromised by the absence of clear reporting of p-values or confidence intervals in correlation and regression analyses. Include appropriate statistical tests (e.g., t-test or ANOVA) to support claims of no significant difference.**

**Response:** Thank you to the review experts for their suggestions and opinions. I believe these suggestions and opinions will contribute to a more scientific presentation of our research work. We have made further revisions on page 6, line 179 of the manuscript (p=0.003, P<0.001).

**7. The abstract is verbose and poorly structured, with overlapping and redundant sentences. Revise the abstract to follow a standard format (Background, Objective, Methods, Results, Conclusion).**

**Response:** Thank you to the review experts for their suggestions and opinions. I believe these suggestions and opinions will contribute to a more scientific presentation of our research work. We have made further revisions on page 2, line 30 of the manuscript.

Rice is one of the world’s most abundant food crops. The amylose content is a key factor affecting the production of high-quality rice. The commonly used method for determining the amylose content is the Iodine-Starch colorimetric method. Due to the limitations of traditional iodine colorimetric methods, this experiment provides a high-throughput method for determining amylose content. Using rice varieties with different genetic backgrounds as experimental materials, the accuracy and practicality of the new method were explored to improve measurement efficiency and simplify the operation process. The results demonstrated a strong correlation (R= 0.9998) between the absorbance values and amylose content, with a relative deviation of less than 1.2%. It showed good repeatability, with a variation range of 0.43% to 1.63%. The experiment takes a short time and can measure 96 samples at once. Therefore, while ensuring the accuracy of experimental results, the high-throughput measurement method improves the efficiency of measuring amylose content. This has important practical significance for early screening and identification of excellent individuals in rice quality genetic breeding programs.

**8. The language throughout the manuscript suffers from frequent grammatical errors, awkward phrasing, and unclear sentence structures. Conduct professional English editing and sentence restructuring.**

**Response:** Thank you to the review experts for their suggestions and opinions. I believe these suggestions and opinions will contribute to a more scientific presentation of our research work. We have made further revisions on the manuscript.

**9. Key experimental variables (e.g., digestion temperature, NaOH concentration) are introduced but not thoroughly tested. Include justification for selected values or conduct sensitivity tests.**

**Response:** Thank you for the experts' review. We have responded to your suggestions and opinions as follows. In the previous work, we found that different researchers have different choices of variable parameters for experiments, such as, Supported by Table 4. Due to the fact that the SSDM scheme is optimized or simplified based on the SDM scheme. The key variables of the experiment were not explored individually, but digestion time and alkaline concentration were discussed accordingly (see attached table). However, in our experiment, we provided experimental data on the wavelength of the selected sample, optimal digestion time, and alkaline concentration.

**References:**

Zhang T, Wang ZR, Mao XC, *et al*. Research progress of genes affecting rice grain quality. *J. Anhui Agric. Sci.* 2023, *51*, 17-22.

Chen, C.; Zhang, Y.F.; Wang, S.H. Study on the determination of amylose content of single rice grain. *Journal of Anhui Agri. Sci.* 2005, *33*, 196-197.

Shao, Y.; Peng, Y.; Mao, B.; Lv, Q.; Yuan, D.; Liu X. Allelic variations of the *Wx* locus in cultivated rice and their use in the development of hybrid rice in China. *PloS One.* 2020, *15*, e0232279.

Zhang C, Zhu J, Chen S, Fan X, Li Q, Lu Y, Wang M, Yu H, Yi C, Tang S, Gu M, Liu Q. *Wx^lv^*, the ancestral allele of rice *Waxy* gene. *Mol Plant*. 2019, *12*, 1157-1166.

Zhang AH, Xu CW, Mo HD, Genetic expression of several quality traits in *indica*-*japonica* hybrids. *Acta Agronomica Sinica* 1999, 25, 401-407.

**10. Table formatting, especially in Tables 1–3, is suboptimal. Improve clarity, define abbreviations, and ensure all values are appropriately labeled with units.**

**Response:** Thank you to the review experts for their suggestions and opinions. I believe these suggestions and opinions will contribute to a more scientific presentation of our research work. We have made further revisions on page 9, line 128 of the manuscript (p=0.003, P<0.001).

**11. The explanation of how SSDM integrates with ELISA technology is vague and potentially misleading. Clarify if ELISA is truly used. If not, remove the term to avoid confusion.**

**Response:** Thank you for the suggestions from the reviewing experts. Your suggestions will be helpful for our work. We have made modifications in the corresponding sections (Page 4 and line 142). The modified content is as follows, utilizing the high accuracy and flexible data collection of microplate reader detection samples, the microplate reader equipment was introduced. In order to avoid any disagreement, the microplate reader was replaced with a microplate reader in the original text, and replace the term ELISA with microplate reader.

**12. SSDM’s reliance on a 96-well plate format and spectrophotometric detection is not novel. Authors should specify what distinguishes SSDM from existing high-throughput assays.**

**Response:** Thank you for the suggestions and opinions from the reviewing experts. We have added the corresponding content on page 11 and line 295 of the manuscript as required (the construction of absorption spectra for standard samples ensures the authenticity of each SSDM measurement result). The reason for the modification is that the content of amylose affects the quality of rice grains. Moreover, in the process of improving rice quality, the requirement for the method of measuring amylose content is to ensure the accuracy of experimental results, while also requiring the experimental method to have the characteristics of simple program, easy operation, and high sample measurement flux. Compared to the SDM method, SSDM has the following important innovations: 1.) By measuring the absorption wavelength of the standard sample, the most suitable measurement wavelength is selected to ensure sample deviation caused by the establishment of the standard curve in the later stage during the operation process; 2.) By utilizing the precision of the microplate reader and the speed of sample detection, the number of sample readings has been increased, while also improving the accuracy of experimental data reading and the convenience of later experimental data recording. In summary, these are the differences between SSDM and existing high-throughput measurement methods.

**13. Figure legends are inadequately described, and essential figures (e.g., method schematic) are missing. Add a schematic comparing SDM and SSDM and revise all figure captions to be self-explanatory.**

**Response:** Thank you for the suggestions and opinions from the reviewing experts. We have made further revisions on the manuscript, and added the corresponding content on page 11 and line 244 of the manuscript as required (Figure 1 and Table 4).

**Figture1** Method schematic. SDM, standard method; SSDM, simplified standard method.

**14. The justification for excluding single-grain analysis is weak. Provide stronger evidence or literature to support this decision, or present it as a limitation.**

**Response:** This is a valuable suggestion. We had considered such materials in the experimental design. However, since the starch content trait in rice endosperm is classified as an endosperm-specific characteristic following triploid genetic laws, previous studies have discussed this aspect. Given the gene dosage effect observed in single grains or small samples of rice, the sample reproducibility was poor, on page 11 and line 260 of the manuscript as required.

In addition, previous studies have shown that when determining the content of amylose, the sample must be fully gelatinized to facilitate the full binding of iodine and starch, thereby reducing errors. In addition, due to differences in grain filling rate during rice growth, uneven quality of individual rice grains may occur. This can lead to inconsistent sample quality during the determination of amylose, ultimately affecting the reproducibility of experimental results. Therefore, combined with the previous research experience, as an improvement of the experimental scheme, this experiment selected Rice noodles of known rice varieties, so as to reduce the error caused by the genetic metrological effect in endosperm, sample quality differences, etc.

**References:**

Zhang T, Wang ZR, Mao XC, *et al*. Research progress of genes affecting rice grain quality. J. Anhui Agric. Sci. 2023, 51, 17-22.

Chen, C.; Zhang, Y.F.; Wang, S.H. Study on the determination of amylose content of single rice grain. *Journal of Anhui Agri. Sci.* 2005, *33*, 196-197.

Zhang, A.H.; Xu, C.W.; Mo, H.D. Genetic expression of several quality traits in *indica*-*japonica* hybrids. *Acta Agronomica Sinica* 1999, 25, 401-407.

**15. References are outdated in several places. Update references with recent publications from the past 5 years.**

**Response:** Thank you for the suggestions from the reviewing experts. Your suggestions will be helpful for our work. We have made modifications in the corresponding sections.

**References:**

Zhang, B.; Qiao, D.; l, Zhao, S.; Lin, Q.; Wang, J.; Xie, F. Starch-based food matrices containing protein: recent understanding of morphology, structure, and properties. *Trends Food Sci Tech*, 2021, *114*, 212-231.

Gibson, T.S.; Solah V.A.; McCleary, B.V. A procedure to measure amylose in cereal starches and flours with concanavalin A *J Cereal Sci*. 1997, *25*, 111-119

Shi, P., Zhao, Y., Qin, F., Liu, K., Wang, H. Understanding the multi-scale structure and physicochemical properties of millet starch with varied amylose content. *Food Chem*. 2023 *1*, 410, 135422.

**16. The discussion section repeats results without deeper interpretation. Expand discussion to include scientific implications, breeding relevance, and future directions.**

**Response:** Thank you for the suggestions from the reviewing experts. Your suggestions will be helpful for our work. We have made modifications in the corresponding sections on page 11 and line 289 of the manuscript as required.

**17. The manuscript does not adequately address performance on unknown or genetically diverse samples. Clarify whether SSDM is applicable to diverse rice germplasm. Acknowledge as a limitation if untested.**

**Response:** Thank you for the suggestions from the reviewing experts. Your suggestions will be helpful for our work. We have made modifications in the corresponding sections. When analyzing an unknown sample, we only need to obtain the content of amylose, regardless of genetic composition. In our experiment, it was mentioned that the examination has a certain genetic background for two purposes. Firstly, it is emphasized that the starch iodine staining method is widely used in rice genetic breeding; Secondly, it proves that the implementation of the experimental plan in the manuscript is evidence-based; At the same time, the detection of specific genetic background materials can indirectly verify the reliability of the SSDM method; Finally, there was a linear relationship between starch-iodine absorbance and amylose content in the experiment. Therefore, we believe that materials with different genetic backgrounds do not affect the performance of SSDM. There was no analysis or discussion in the original manuscript. (on page 11 and line 280)

**18. The conclusion overstates the practical implications of SSDM. Moderate the conclusion and align it with the actual findings and scale of validation.**

**Response:** Thank you for the suggestions from the reviewing experts. We have made corresponding modifications in the conclusion section of the manuscript on page 11 and line 241. The content that needs to be modified is as follows: Previous research has shown that the content of amylose affects the taste, appearance, and nutritional quality of rice. However, in the genetic breeding of rice quality improvement, due to the large sample size in the early stage, the advantages of SSDM itself have improved the efficiency of measuring amylose content, accelerated the screening process of early target traits, and its good repeatability and ease of operation have made SSDM widely applicable. Therefore, compared with traditional SDM technology, SSDM has better advantages in the preliminary screening of rice quality traits.

**References:**

Zhang, B.; Qiao, D.; l, Zhao, S.; Lin, Q.; Wang, J.; Xie, F. Starch-based food matrices containing protein: recent understanding of morphology, structure, and properties. *Trends Food Sci Tech*, 2021, 114: 212-231.

Zhou, H.; Xia, D.; Zhao, D.; Li, Y.; Li, P.; Wu, B.; Gao, G.; Zhang, Q.; Wang, G.; Xiao, J.; et al. The origin of *Wx^la^* provides new insights into the improvement of grain quality in rice. *J Integr Plant Biol*. 2021, *63*, 878-888.

Shao, Y.; Peng, Y.; Mao, B.; Lv, Q.; Yuan, D.; Liu X. Allelic variations of the *Wx* locus in cultivated rice and their use in the development of hybrid rice in China. *PloS One.* 2020, *15*, e0232279. .

Wu, Y.; He, Y.; Sretenovic, S.; Liu, S.; Cheng, Y.; Han, Y.; Liu, G.; Bao, Y.; Fang, Q.; Zheng, X.; et al. CRISPR-BETS: A base editing design tool for generating stop codons. *Plant Biotechnol J.* 2021, *20*, 499-510.

Li, Q.; Liu, X.; Zhang, C.; Jiang, L.; Jiang, M.; Zhong, M.; Fan, X.; Gu, M.; Liu, Q. Waxy rice soluble starch synthase I: allelic variation, expression, function, and interaction with. *Front. Plant Sci.* 2018, *13*, 1591.

Tian Z, Qian Q, Liu Q, Yan M, Liu X, Yan C, *et al*. Allelic diversities in rice starch biosynthesis lead to a diverse array of rice eating and cooking qualities. Proc. Natl. Acad. Sci. USA. 2009, 106, 21760-21765.

Tetlow, I.J.; Beisel, K.G.; Cameron, S.; Makhmoudova, A.; Liu, F.; Bresolin, N.S.; Wait, R.; Emes, M.J. Analysis of protein complexes in wheat amyloplasts reveals functional interactions among starch biosynthetic enzymes. *Plant Physiol.* 2008, *146*, 1878-1891.

**19. Although the SSDM exhibits low variability in repeated measures, the magnitude of improvement over SDM is marginal. Quantify the gains in throughput, time, or cost explicitly.**

**Response:** Thank you to the review experts for their suggestions and opinions. I believe these suggestions and opinions will contribute to a more scientific presentation of our research work. We have made further revisions on page 10, line 244 of the manuscript. (As see Table 4)

**20. No limitations section is included. Add a section discussing limitations (e.g., dependency on known genotypes, lab setup requirements, variability).**

**Response:** Thank you to the review experts for their suggestions and opinions. I believe these suggestions and opinions will contribute to a more scientific presentation of our research work. We have made further revisions on page 11, line 267 of the manuscript.

This is a valuable suggestion. We had considered such materials in the experimental design. However, since the starch content trait in rice endosperm is classified as an endosperm-specific characteristic following triploid genetic laws, previous studies have discussed this aspect. Given the gene dosage effect observed in single grains or small samples of rice, the sample reproducibility was poor.

In addition, previous studies have shown that when determining the content of amylose, the sample must be fully gelatinized to facilitate the full binding of iodine and starch, thereby reducing errors. In addition, due to differences in grain filling rate during rice growth, uneven quality of individual rice grains may occur. This can lead to inconsistent sample quality during the determination of amylose, ultimately affecting the reproducibility of experimental results. Therefore, combined with the previous research experience, as an improvement of the experimental scheme, this experiment selected Rice noodles of known rice varieties, so as to reduce the error caused by the genetic metrological effect in endosperm, sample quality differences, etc.

**Reference:**

Liu JL, Shang B, Xing XT, Zhang D, Chang L, Sun H, Duan XL. Comparison of four methods for the determination of the amylose content in foxtail millet. Food Science, 2023, 44(12): 217-224.

**21. The authors' response to prior reviewer critiques is extensive but lacks depth in several places. Responses should go beyond wording edits and address core methodological concerns.**

**Response:** Thank you to the review experts for their suggestions and opinions. I believe these suggestions and opinions will contribute to a more scientific presentation of our research work. We have made further revisions on page 11, line 267 of the manuscript, such as Q14 and Q17.

**22. Claims about SSDM's high throughput would benefit from benchmarking against commercial kits or rapid analytical platforms. Compare SSDM with commercial or literature methods in terms of speed, sensitivity, and cost.**

**Response:** Thank you to the review experts for their suggestions and opinions. I believe these suggestions and opinions will contribute to a more scientific presentation of our research work. We have made further revisions on page 10, line 244 of the manuscript.

**Table 1** Comparison of different methods for determining the content of amylose

| Method Schematic | | SDM | SSDM |
| --- | --- | --- | --- |
| sample preparation | Sample uniform | 80-100 mesh sieve | 100 mesh sieve |
|  | Sample weight | 100 ± 0.5 mg | 20 ± 0.05 mg |
|  | Dispersion liquid | 1.0 mL anhydrous ethanol | 0.2 mL anhydrous ethanol |
| dispersion and gelatinization | Pasting temperature | 100 ℃ | 60 ℃ |
|  | Pasting time | 10 mins | 2 h |
|  | Gelatinization container | 100 mL conical flask | 2.0 mL centrifuge tube |
| Color reaction | Sample dosage | 5.0 mL | 0.1 mL |
|  | 1N NaAC | 1.0 mL | 0.2 mL |
|  | 0.2% I_2_-KI | 2.0 mL | 0.2 mL |
|  | distilled water | 92.0 mL | 9.5 mL |
|  | testing vessels | 1 cm cuvette | 96 cell transparent plate |
|  | test equipment | a spectrophotometer | microplate reader |
|  | detection wavelength | 720 nm | 620 nm |
| Test sample quantities | 2 h per person | 12 samples | 96 samples |

**23. SSDM’s reliance on microplate reader and shaker may limit its use in field labs. Acknowledge instrumentation limitations and discuss potential adaptations.**

**Response:** Thank you to the review experts for their suggestions and opinions. I believe these suggestions and opinions will contribute to a more scientific presentation of our research work. We have made further revisions on page 11, line 303 of the manuscript

**24. The acronym “SSDM” includes ELISA, which is not implemented in the actual method. Correct acronym expansion or remove misleading reference to ELISA.**

**Response:** Thank you to the review experts for their suggestions and opinions. I believe these suggestions and opinions will contribute to a more scientific presentation of our research work. We have made further revisions on page 4, line 142 of the manuscript.

**25. The SSDM method appears suitable for preliminary screening but lacks validation across diverse use cases. Clearly define current validation scope and outline required future studies for broader adoption.**

**Response:** Thank you to the review experts for their suggestions and opinions. I believe these suggestions and opinions will contribute to a more scientific presentation of our research work. We have made further revisions on page 11, line 289 of the manuscript. However, in the genetic breeding of rice for improving rice quality, due to the large sample size in the early stage. Due to the advantages of SSDM, such as improved efficiency, good repeatability, and ease of operation in the determination of amylose content. Therefore, compared with traditional SDM technology, SSDM has better advantages in the preliminary screening of rice quality traits.

**26. In Table 1, the authors present RSD ranges in descending order (e.g., “2.5410.53”). This is unconventional; standard scientific reporting typically presents RSD ranges in ascending order, please correct it.**

**Response:** Thank you to the review experts for their suggestions and opinions. I believe these suggestions and opinions will contribute to a more scientific presentation of our research work. We have made further revisions on page 8, line 221 of the manuscript.
