# Supplementary material for: A high-throughput method for determining the amylose content of rice
Source: PLoS One. 2025 Nov 25;20(11):e0336600. doi: 10.1371/journal.pone.0336600 (PMC12646410; doi:10.1371/journal.pone.0336600)
Supplement: Attachment — Submitted filename: Responses to academic reviews 1013.docx [file pone.0336600.s010.docx]

**Responses to Academic Editors' comments**

I sincerely thank you and the academic editors for their comments and put forward very constructive suggestions and opinions to us. We have carefully reviewed and revised the manuscript. We believe that under the guidance of our beloved editors and review experts, we can give good and appropriate responses to all the comments listed below through our careful modification. The following is a list of the main revisions based on the opinions of editors and reviewers:

1. According to the comments of editors and reviewers, we highlighted the purpose of the research and the innovative aspects of our work in the manuscript to facilitate readers' understanding of our work.
2. According to the reviewer's comments, we modified and corrected the content and wording of the manuscript.
3. We have carefully revised the manuscript, including correcting typing errors, adding content to explain the data in the chart, and modifying all texts according to the concerns of the reviewers. Please refer to the main revised manuscript for details.

Through our careful efforts to revise and improve, we hope that the revised version can meet the requirements and make the manuscript published in the form of *PloS One*. Thank you again for your wonderful comments and your valuable time for our manuscript. We sincerely look forward to seeing our working manuscript in your journal.

**Academic Editors' and Reviewers' comment on ONE-D-24-60263R2**

**RESPONSES TO ACADEMIC REVIEWER #1**

**Comments and Suggestions, and Journal Requirements for Authors**

1. **If the reviewer comments include a recommendation to cite specific previously published works, please review and evaluate these publications to determine whether they are relevant and should be cited. There is no requirement to cite these works unless the editor has indicated otherwise.**

**Response：**We sincerely appreciate the valuable suggestions and comments from the reviewers. These constructive recommendations will significantly contribute to improving our manuscript. We have carefully addressed all the reviewers' comments in the revised version. Specific modifications include citing previous research results and deleting literature with weak correlation (on page 17).

2. **Please review your reference list to ensure that it is complete and correct. If you have cited papers that have been retracted, please include the rationale for doing so in the manuscript text, or remove these references and replace them with relevant current references. Any changes to the reference list should be mentioned in the rebuttal letter that accompanies your revised manuscript. If you need to cite a retracted article, indicate the article’s retracted status in the References list and also include a citation and full reference for the retraction notice.**

**Response：**We sincerely appreciate the editors' valuable suggestions and comments. These constructive recommendations have significantly contributed to improving our manuscript. Accordingly, we have implemented the following modifications: We accept suggestion of the reviewing experts. At the suggestion of editors', we carefully reviewed relevant literature and replaced reference 11-13, 21-23, and 26 on page 18, line 349 of the original manuscript with the corresponding new reference. We believe these revisions will enhance the manuscript's structural clarity and content completeness.

**RESPONSES TO ADDITIONAL ACADEMIC REVIEWERS #2**

**Comments and Suggestions, and Journal Requirements for Authors**

1. **Clarify and strengthen the novelty of SSDM compared to existing amylose determination methods (e.g., NIR, other rapid colorimetric adaptations). Explicitly state the research gap that SSDM addresses.**

**Response：**We sincerely appreciate the editors' valuable suggestions and comments. These constructive recommendations have significantly contributed to improving our manuscript. Accordingly, we have implemented the following modifications: We accept the suggestion of the reviewing experts. At the suggestion of editors', we carefully reviewed relevant literature and replaced the content on page 14, lines 253-260 of the original manuscript with the corresponding updated content. We believe these revisions will enhance the manuscript's structural clarity and content completeness.

2. **Revise the methods section for reproducibility. Remove all formatting artifacts and duplicated text, and clearly state whether replicates are biological or technical. Ensure the protocol is transparent enough for replication.**

**Response：**We sincerely appreciate the valuable suggestions and comments from the editors and reviewers. These constructive recommendations will significantly contribute to improving our manuscript. We have carefully addressed all the reviewers' comments in the revised version. Specific modifications include: Each sample underwent three technical replicates, improving repeatability in the method section (page 11, lines 211-225). And remove all formatting artifacts and duplicate text from the manuscript.

3. **Provide proper statistical analysis beyond RSD values. Apply appropriate tests (e.g., ANOVA, t-tests) to support claims of superiority and report corresponding significance levels.**

**Response：**We sincerely appreciate the valuable suggestions and comments from the editors and reviewers. These constructive recommendations will significantly contribute to improving our manuscript. Statistical analysis was conducted in Tables 1-3 on page 11, line 211 of the manuscript.

4. **Expand the limitations section to acknowledge the small validation set (six rice varieties), the dependency on specialized instruments such as a microplate reader, and the lack of validation across broader varietal or environmental conditions.**

**Response：**We sincerely appreciate the valuable suggestions and comments from the editors and reviewers. Corresponding modifications were made on page 15, line 279 of the manuscript.

5. **Moderate the conclusions. Restrict claims to what is directly supported by the dataset and avoid overstating applicability or adoption potential.**

**Response：**We sincerely appreciate the valuable suggestions and comments from the editors and reviewers. Corresponding modifications were made on page 16, lines 284-302 of the manuscript. We believe these revisions will enhance the manuscript's structural clarity and content completeness.

6. **Correct all terminology inconsistencies (e.g., misuse of ELISA terminology, inconsistent references to “starch-iodine” vs “amylose-iodine”) and ensure uniform usage throughout.**

**Response：**We sincerely appreciate the valuable suggestions and comments from the editors and reviewers. Corrected the relevant terminology in the manuscript according to the suggestions and requirements of academic editors.
